# Edge computing for detection of ship and ship port from remote sensing images using YOLO

**DOI:** 10.3389/frai.2025.1508664

**Published:** 2025-02-06

**Authors:** Vasavi Sanikommu, Sai Pravallika Marripudi, Harini Reddy Yekkanti, Revanth Divi, R. Chandrakanth, P. Mahindra

**Affiliations:** ^1^Department of Artificial Intelligence and Data Science, Velagapudi Ramakrishna Siddhartha Engineering College, Vijayawada, India; ^2^Department of Space, Advanced Data Research Institute (ADRIN), Hyderabad, India

**Keywords:** ship detection, ship-port detection, You Only Look Once (YOLO), edge computing, deep learning, R-CNN, satellite imagery

## Abstract

In marine security and surveillance, accurately identifying ships and ship ports from satellite imagery remains a critical challenge due to the inefficiencies and inaccuracies of conventional approaches. The proposed method uses an enhanced YOLO (You Only Look Once) model, a robust real-time object detection method. The method involves training the YOLO model on an extensive collection of annotated satellite images to detect ships and ship ports accurately. The proposed system delivers a precision of 86% compared to existing methods; this approach is designed to allow for real-time deployment in the context of resource-constrained environments, especially with a Jetson Nano edge device. This deployment will ensure scalability, efficient processing, and reduced reliance on central computing resources, making it especially suitable for maritime settings in which real-time monitoring is vital. The findings of this study, therefore, point out the practical implications of this improved YOLO model for maritime surveillance: offering a scalable and efficient solution to strengthen maritime security.

## 1 Introduction

Detection of ships and ship ports in satellite imagery is of high importance to numerous domains such as maritime surveillance, environmental monitoring, and global security. The accuracy and efficiency in the detection of maritime activities are of paramount importance when dealing with challenges such as illegal fishing, smuggling, and piracy as well as for the proper management of ports and navigation. However, such traditional detection methods lack precision and cannot be scaled enough to monitor massive maritime regions in diverse environmental conditions, therefore leaving gaps within monitoring and responding capabilities.

The ability to break the limitations of existing detection models has been offered through deep learning algorithms, specifically object detection models. Currently, however, techniques exist, and while these are effective up to a point, they present various problems with changing environmental conditions, cluttered background scenes, and limited computational resources. Thus, resolving these matters is crucial in making improvements in maritime surveillance and maintaining maritime activities as safe and secure as possible. Maritime operations are crucial for global trade, transportation, and security. It must be monitored to prevent illegal activities and protect the marine ecosystem. Satellite images can be a powerful monitoring tool for large maritime areas. They provide an overview of all ship movements and port activities, but manual analysis of satellite imagery is labor-intensive and time-consuming, highlighting the need for automated detection systems to improve the efficiency and accuracy of maritime surveillance efforts.

Edge computing is a transformative way of enabling real-time insights on maritime monitoring. It reduces the latency, optimizes bandwidth usage, and improves the systems' reliability by processing data as close to its source. This localized processing ensures operational continuity while ensuring that data is not exposed excessively, thus enhancing privacy and security. Advanced detection systems deployed on edge devices such as Jetson Nano could prove highly effective for fulfilling the computational requirements of maritime surveillance without any compromise on efficiency. The advantages of edge computing have drawbacks like resource constraints on the available power, memory, and storage capacity. Moreover, integration complexities resulting from a large variety of devices and systems call for robust interoperability solutions. Managing data synchronization between systems and addressing security vulnerabilities such as the risk of physical tampering, malware attacks, is needed to unleash the true edge-computing power. For this research, by solving the associated challenges, a dependable, secure and efficient solution will be available for edge-based ship and port detection at the ship end; this can be of immense importance for maritime security surveillance.

### 1.1 Objectives of the proposed study

The objectives of this research work are:

- To create a dataset with annotated images of ship and ship ports.
- To automate the detection of ship ports from high-resolution satellite imagery using an enhanced YOLO model.
- To evaluate the accuracy of the enhanced object detection model to ensure its reliability for real-time applications.
- To deploy the detection model on a Jetson nano edge device in order to decrease the power consumption.

### 1.2 Contributions

- Created a dataset consisting of satellite imagery for ship-ports belonging to Australia and India.
- Developed an enhanced YOLO model with features such as multi-scale object detection for ship port detection.
- Deployed the model on an edge device for real-time processing and found that it significantly reduced the latency from cloud computing (10–100 ms) to (1–10 ms) on an edge device.

### 1.3 Organization

The paper is organized as follows: Section 2 reviews existing ship and object detection research. Section 3 outlines the proposed architecture and methodologies. Section 4 presents the evaluation and results. Section 5 concludes with recommendations for future work.

## 2 Literature review

This section presents existing techniques for ship and ship port detection, identify their limitations, and propose a novel approach utilizing deep learning algorithms, specifically enhanced YOLO (Girshick et al., 2014), to address these challenges. The suggested model's performance is tested using real-world satellite imagery datasets and demonstrates its potential for enhancing maritime surveillance capabilities. This paper aims to contribute to developing more effective and efficient methods for monitoring maritime traffic and safeguarding our oceans.

For identifying ships in satellite photos (Patel et al., 2022a), evaluated the YOLOv3, YOLOv4, and YOLOv5 algorithms. Considering the Shipsnet and Airbus Ship Challenge datasets as the basis for evaluation, it has been found that YOLOv5 has the highest detection accuracy of 99%, in contrast to 98% and 97% for YOLOv4 and YOLOv3. YOLOv5 is a more accurate solution for ship detection jobs even though it is slower than YOLOv3. The research emphasizes the significance of choosing an appropriate algorithm for precise ship detection and demonstrates the practical uses of YOLO-based algorithms, particularly YOLOv5, in environmental monitoring and maritime surveillance.

The optimum ship recognition algorithm for small and gathering ships was developed by Zhang et al. (2019) using high-resolution remote sensing data. Determine possible regions of interest (ROIs) that contain ships by dividing areas with and without water using a coarse-to-fine method. To increase the recognition of small clustered ships, they developed the Faster-R-CNN framework based on VGG16, and used the R-CNN method to identify ships inside the ROIs. Higher recall and accuracy were obtained with their enhanced Faster-R-CNN in comparison to other methods. Future research on the topic should look into traditional preprocessing methods including LBP, SML, PCA classifier variants, and Gaussian local descriptors in order to increase ROI recall.

To improve marine surveillance accuracy, Patel et al. (2022b) described a ship detection method that integrates the Graph Neural Network (GNN) and YOLOv7 deep learning frameworks. The approach is to increase knowledge about ships' presence in harbor environments. After experimenting with hyperparameters including learning rate, batch sizes, and optimization selection, it has been discovered that Adam's optimization outperforms earlier YOLOv7 iterations with a success rate of 93.4%. The system obtained over 90% ship classification accuracy using the High-Resolution Satellite Image Dataset (HRSID), which is derived from synthetic aperture radar.

Ngo et al. (2023) have addressed the urgent need for improved methodology in maritime surveillance with their groundbreaking study on deep variational information bottleneck approaches for image-based ship detection. Their system attempts to increase the robustness and accuracy of ship detection algorithms in satellite data by combining deep learning with the concepts of variational information bottlenecks. The described model emphasizes how crucial it is to use cutting-edge computational methods in order to efficiently manage the enormous amounts of data coming from contemporary satellite platforms. The authors prove the accuracy of their approach in ship identification through empirical tests.

A deep learning method for automatic ship detection in satellite imagery was presented by Stofa et al. (2020), who emphasized the need for high-precision ship detection for national security. The primary convolutional neural network classifier employed was the DenseNet architecture and optimized the performance by adjusting hyperparameters such as batch size, optimizer selection, and learning rate. With the Adam optimizer, the experimental results on the Kaggle Ships dataset showed an impressive success rate of over 99.75% and a learning rate of 0.0001. The study highlights the possibility for more improvement through the investigation of different convolutional neural network structures, and the ability of DenseNet to get a ship classification accuracy exceeding 90% when the ideal hyperparameter values are found.

Shen et al. (2022) described a multi-class geospatial object detection technique that is essential for applications like military surveillance and urban planning and was created for large-scale remote sensing data. By producing multi-volume YOLOv4 through CNN pruning, their method achieves a balance between accuracy and efficiency. By integrating the Manhattan Distance Intersection of Union (MIOU) loss function, they enhance accuracy. Additionally, they recommend the use of shortened Non-Maximum Suppression (NMS) to omit false positives from shortened targets. Evaluations on the DOTA and DOTA v2 datasets show superior performance; mAP and FPS exceed 77.3/35 and 61.0/74, respectively.

Qiu et al. (2017) described a novel approach to improve occluded item detection in high-resolution remote sensing images: automating the creation of per-pixel classification maps (PCMs). The difficulty of effectively recognizing objects is addressed by the suggested study, especially in intricate settings with frequent occlusions. The goal of automating PCM production is to increase the speed and accuracy of remote sensing picture processing by accelerating the detection of occluded objects. The described technology overcomes the drawbacks of human methods by using sophisticated computational algorithms, enabling quick and accurate detection of obscured objects in high-resolution pictures. The method's efficacy has been confirmed through empirical validation, providing insightful information and a workable approach to improve remote sensing picture interpretation jobs. This work marks a substantial development in automated phase change for occluded object detection, benefiting researchers and practitioners in remote sensing and geoscience fields.

Deng et al. (2018) described a deep CNN-based method for multi-class object classification in remote sensing images, addressing problems with sparse annotations and scale variability. Their methodology includes a redesigned feature extractor and two sub-included networks: a Multi-scale Object Proposal Network (MS-OPN) which generates object-like features and regions with varying scales, and An Accurate Object Detection (AODN) for fused feature maps for object recognition. Several datasets have been evaluated, demonstrating superior performance in recognizing items with scale variability and densely packed small-size objects.

Yan et al. (2019) describe a novel approach to improve multi-class item recognition in remote sensing images. By employing an IoU-based weighted loss function during training and introducing the Class Aspect Ratio Constrained Non-Maximum Suppression (CARC-NMS) method for post-processing, the presented methodology reduces false positives and increases detection accuracy. The DOTA dataset was used for thorough testing, and the system produced state-of-the-art object detection results while outperforming baseline networks. The study offers suggestions for future research topics, such as examining Scale Normalization for Image Pyramids (SNIP) techniques to improve network performance and addressing issues like oriented bounding boxes. It also highlights the study's versatility across different spatial resolutions of remote sensing data.

Zhang et al. (2020) described a novel deep-learning network that detects ships in synthetic aperture radar (SAR) data. The purpose of HyperLi-Net design is to yield an accurate and fast ship identification process. This research paper highlights the importance of effective ship detection in SAR imaging for numerous applications like navigation and maritime surveillance among others. Instead, what the paper talks about is how this network uses SAR data to resolve problems faced by previous techniques thereby enhancing detection performance. The authors conduct a thorough analysis of the functionality of HyperLi-Net when it comes to ship detection tasks while comparing its advantages with other techniques. It makes an important contribution in the field of remote sensing technology for detecting vessels more specifically within reconfiguring SAR images.

### 2.1 Research gaps

- Limited discussion on addressing challenges posed by complex shapes and backgrounds in ship port detection methods.
- A gap in understanding the proposed approach's real-time performance and accuracy compared to existing methods.
- Lack of clarity on evaluation metrics used to compare the proposed ship port detection method with others.
- Potential for future research to optimize the proposed method for real-time performance.
- Opportunity to enhance ship port detection systems by integrating multimodal data sources for improved accuracy and reliability.

## 3 Architecture and methods

**Figure 1:**
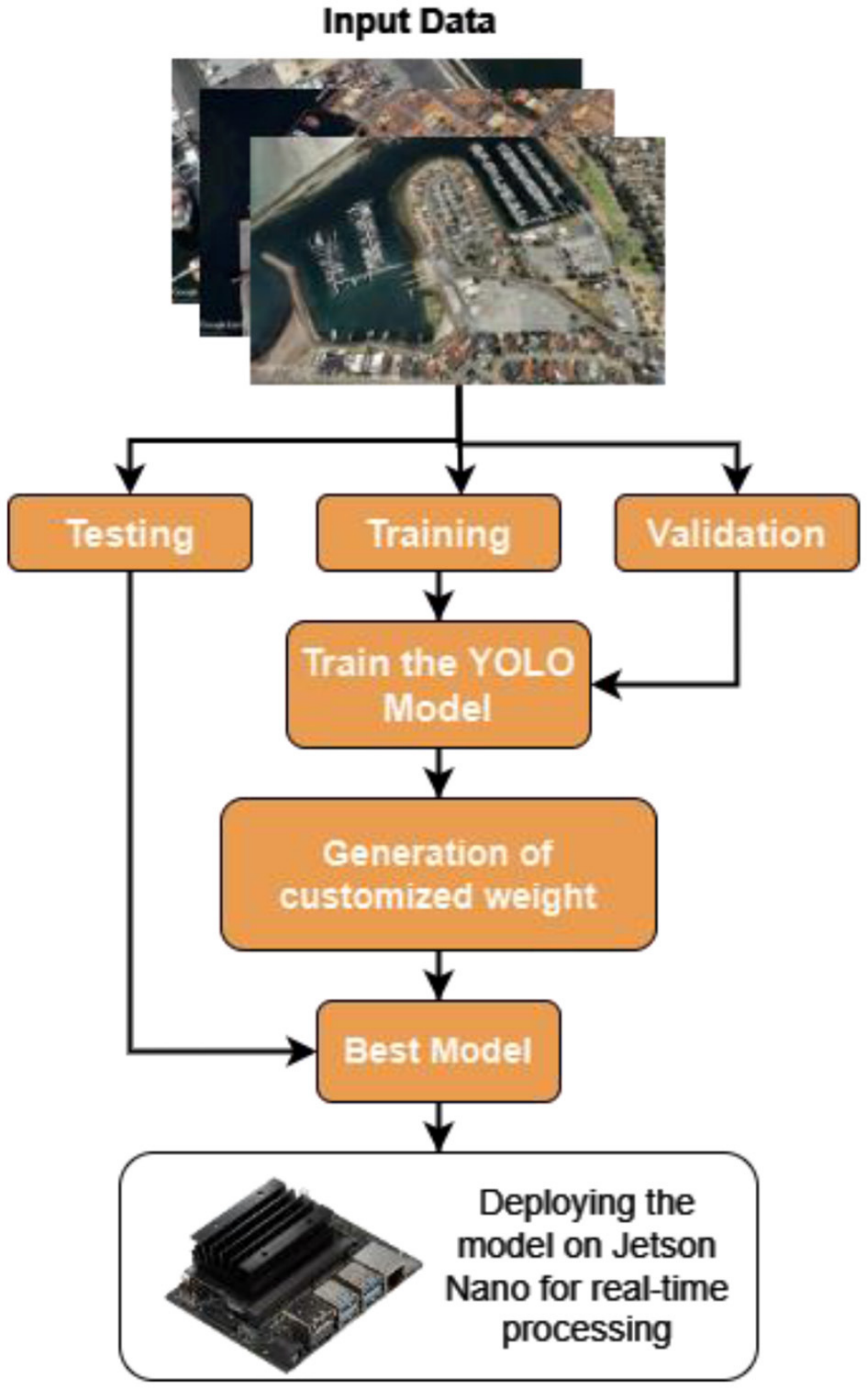
Proposed model architecture.

**Figure 2:**
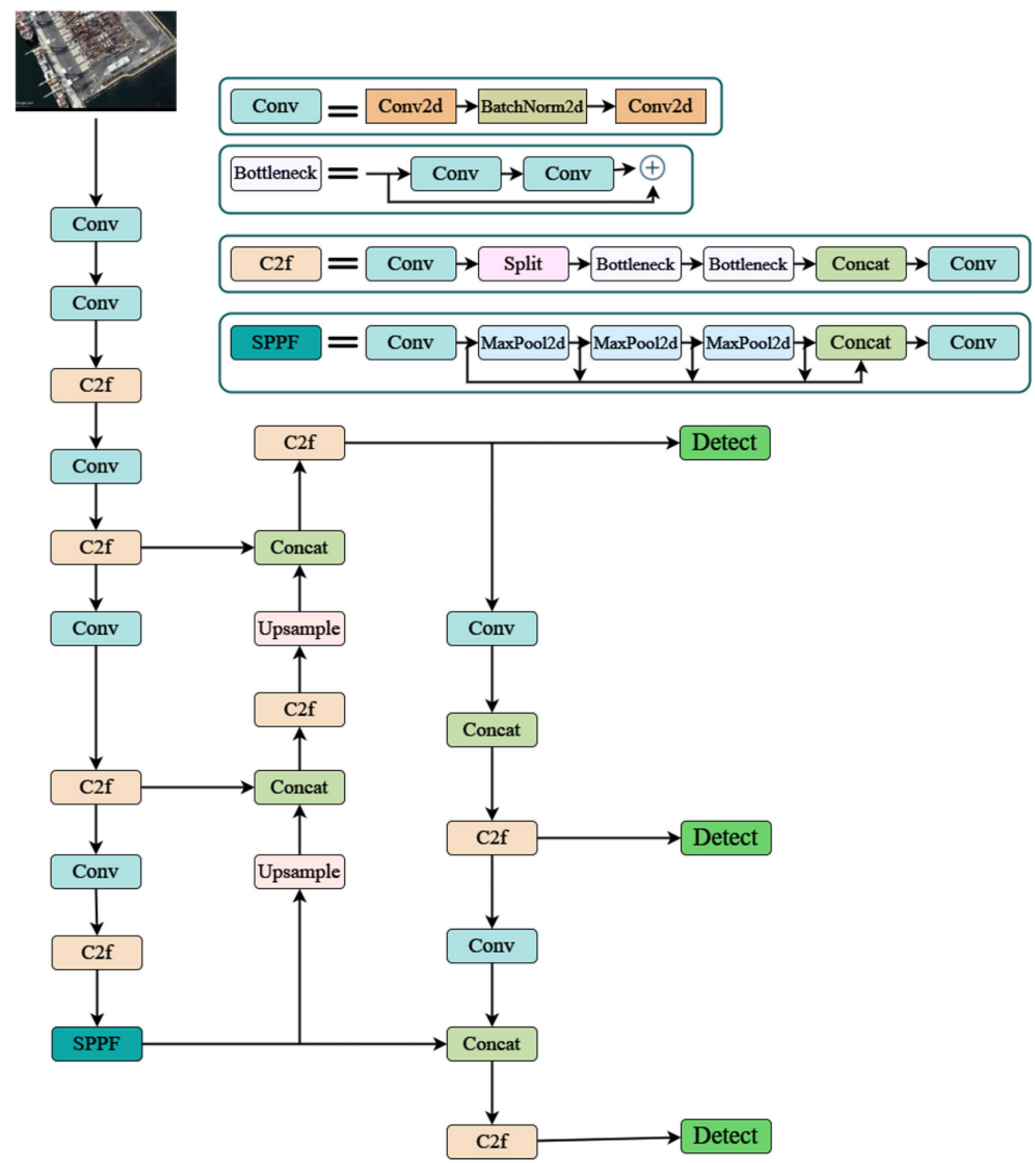
Enhanced YOLO architecture.

The architecture of the model used the datasets, and the procedure used to carry out all the tasks are shown in Figure 1. Figure 2 defines the proposed YOLO architecture used for the method. The enhanced YOLO architecture comprises 22 layers and the efficient layers such as Bottleneck, Conv2d are designed efficiently to detect objects in images. This study uses the unique characteristics of each layer and tailors them for ship and port detection tasks.

**Convolutional layers (Conv, Conv2d):** Convolutional layers serve as the backbone of feature extraction in the enhanced YOLO architecture. These layers learn to identify low-level features such as edges, lines, and corners, which are crucial for detecting ships and ports. In this study, convolutional layers enable the model to recognize specific shapes of ships, and container stacks in ports forwarding to an accurate detection.

**Bottleneck:** This feature in YOLO ensures that the model learns the complex features from low-level features. When the data is processed, the various layers in it with any number of filters ensure that the model can identify the wide range of patterns specific to the ships and ports. This feature allows the model to categorize different types of ships, the containers, and other structures within the ports with a higher accuracy.

**BatchNorm2d:** This is responsible for maintaining and increasing the training process of the YOLO model. It promotes toward a faster convergence and increase the models performance on the unseen data.

**MaxPool2d:** These layers are applied to increase the performance efficiency and to reduce the overfitting by lowering the dimensionality of feature maps. This layer helps in focusing on the important features in the input images and omitting the background details.

**Custom layer (C2f):** The enhanced model is specifically fine-tuned on anchor box prediction and feature transformation. This optimizes the models detection in ship and ship ports. It advances the models efficiency further in outlining required and specific features that are essential for ship and ship port detection.


(1) $$\begin{array}{l} Anchor\;box\;width=image\text{\_}width\times e_{w} \end{array}$$

(2) $$\begin{array}{l} Anchor\;box\;height=image\text{\_}height\times e_{h} \end{array}$$

Furthermore the Up-sample layer helps in detecting small ships by increasing the feature-map resolution by giving bounding boxes around the predicted image. With the SPP layers identifying the features at different scales, the detect layer learns all these features to differentiate between ships, ports, containers and various other elements. It generates bounding box proposals at last around the predicted images and class probabilities based on the feature maps.

**Bounding box prediction (Zheng et al.**, **2020****):**


(3) $$\begin{array}{l} x_{center}=\sigma \left(t_{x}\right)+c_{x} \end{array}$$

(4) $$\begin{array}{l} y_{center}=\sigma \left(t_{y}\right)+c_{y} \end{array}$$

(5) $$\begin{array}{l} width=p_{w}\times e^{tw} \end{array}$$

(6) $$\begin{array}{l} height=p_{h}\times e^{th} \end{array}$$

**Figure 3:**
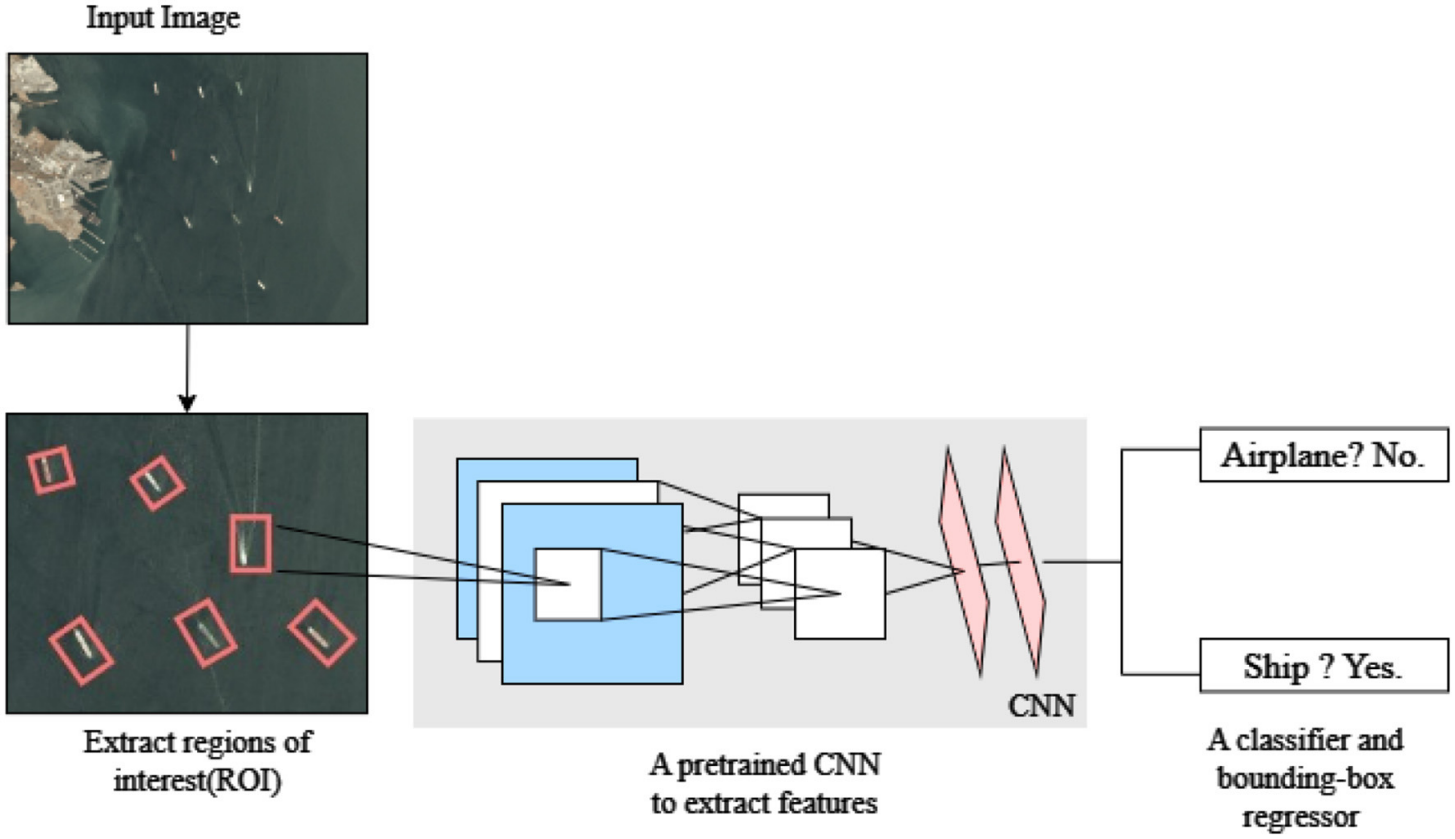
R-CNN model architecture.

The overall architecture, as illustrated in Figure 3, showcases the sequential flow of operations, from initial feature extraction to final object classification and localization, culminating in precise ship detection within input images. While employing this the model used Selective Search which over-segments images using a superpixel algorithm and merges them based on color, texture, size, shape, and meta-similarity measures. Specifically for ship detection, R-CNN (Region-Based Convolutional Neural Network) is utilized to effectively identify and localize ship structures within images. The model's architecture begins with convolutional layers, which serve as the initial stage for feature extraction, capturing low-level features like edges and textures that are relevant to ship structures.

This multi-stage approach utilizes Region Proposal Networks which generates bounding box proposals over the regions which enhances the accuracy of ship detection. Various relevant features are extracted from these proposed regions using RoI pooling, enhancing the precision and accuracy of ship detection. Onward the RoI pooling stage, object classification and bounding box regression are performed through FC (Fully Connected) layers. These layers help in determining the class of the detected object and adjusting the coordinates for precise localization of ships within the images. Through this layer-by-layer feature extraction, the proposed model achieves a high level of accuracy in efficiently detecting ship structures, making it suitable for detecting ships within complex images.

As shown in Table 1 hyper parameters have been adjusted within the proposed model that suits to the application considered in this work. For instance, the proposed model incorporated three channels for the input image which is very crucial in the accurate detection and it further differentiates between different objects of interest in the input images. Opting for a stride of two in the proposed model stands as a pivotal step in the faster processing of the input images which in turn gave a good balance between feature extraction and computational efficiency. While maintaining a standard size for the input kernel, the proposed model employed 64 initial filters to ensure that the model learns complex patterns and features from the satellite images. Also, 22 layers are considered in the bottleneck layer so as to detect the ship ports accurately. Even though this is slightly higher than the existing that is used in YOLOv7 and VGG models, this helped to detect small ship objects in ship ports. Overall the proposed model extends the capability of detecting ship and ship ports as well by making various adjustments to the parameters.

**Table 1:** Comparison of proposed ensemble model w.r.t various parameters used.

**Parameters**	**YOLOv7 model (Patel et al., 2022b)**	**VGG model (Zhang et al., 2019)**	**Proposed enhanced YOLO**
Channels in the input image	1	3	3
Form of the input image	224 × 224 × 1	224 × 224 × 1	648 × 648 × 3
Strides	2	1	2
Size of the input kernel	3 × 3	3 × 3	3 × 3
Number of initial filters	64	32	64
Pooling type	Average Pooling	Max Pooling	Max Pooling
Dimensions of pooling at each layer	2 × 2	2 × 2	2 × 2
No. of layers	18	20	22
Channels in the resulting image	1	3	3

### 3.1 Methodology

This section provides the specific design for the proposed detection system using R-CNN and enhanced YOLO. First, the description will go through the dataset used and then the algorithms are introduced and evaluated.

#### 3.1.1 Dataset

**Figure 4:**
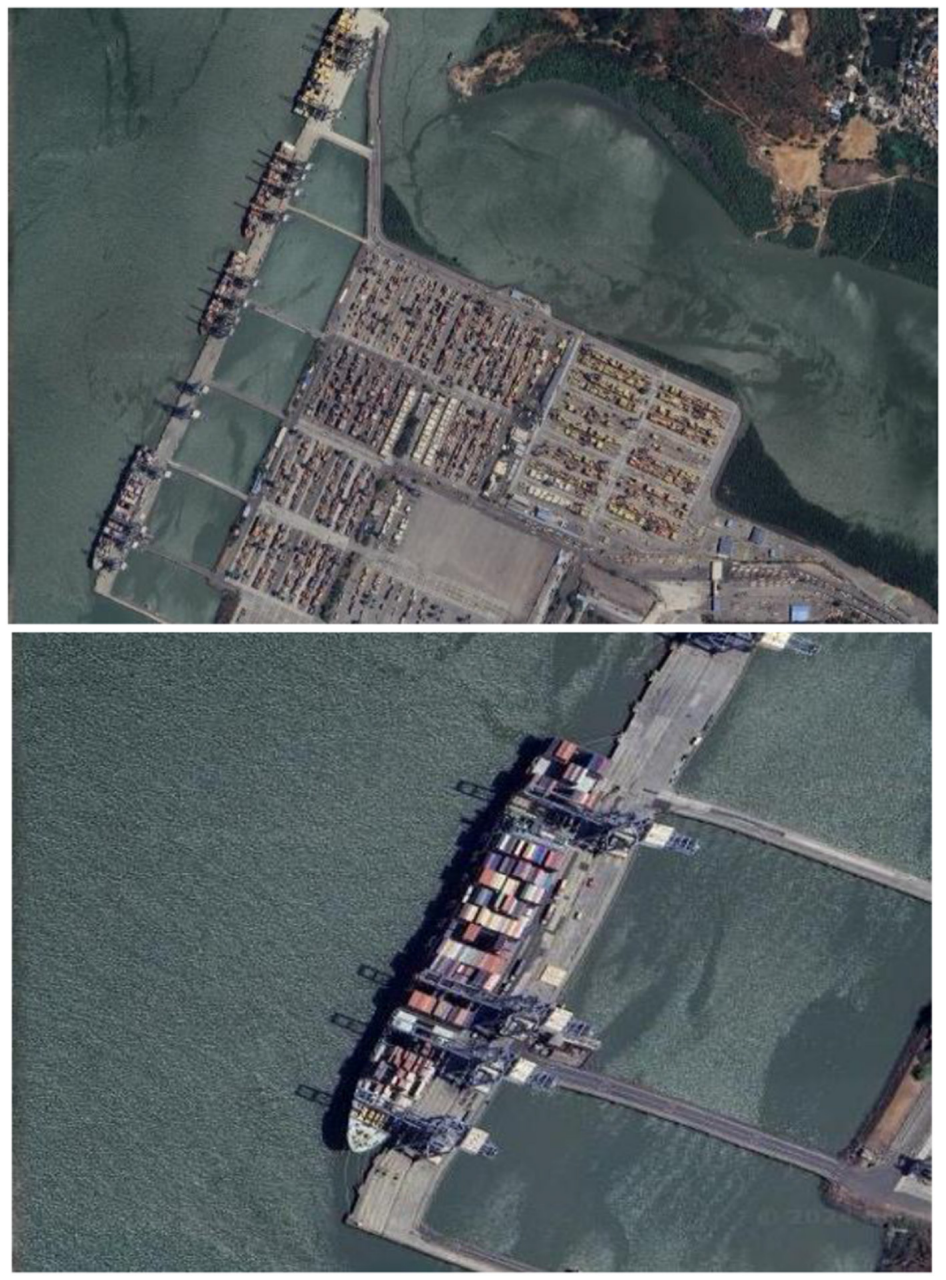
Sample dataset images.

The dataset is manually curated by collecting the various ship and port images from the SAS planet and Google Earth with a current resolution of 3 m and some with a resolution of high-definition (HD) (GeoJamal, 2024). These are specifically chosen as they help with the satellite imagery analysis. These datasets cover a diverse range of geographical locations globally, including coastal areas, major ports, and shipping lanes. Additionally these include various environmental conditions such as cloudy weather, clear sky, and different lighting conditions like daytime and nighttime. Figure 4 presents the satellite images of both ship and a ship port.

#### 3.1.2 Dataset size

The dataset initially consists of approximately 865 RGB images, each with a measuring of 80 × 80 pixels. These are a combination of both ship and ship ports. To standardize image representation and ensure fair comparisons, the dataset underwent preprocessing steps. As described in Cai et al. (2016) and Zhang et al. (2020) the standard preprocessing steps were resizing, and color conversion. This step includes resizing all the images to 48 × 48 pixels and the normalizing step falls within the range of 0 to 1.

#### 3.1.3 Data augmentation

To enhance the robustness and predictability of the model we ensured that various augmentation techniques are performed on the data such as Horizontal flipping, Vertical flipping, Rotation, Scaling, Cropping etc. By integrating all these augmentation techniques, the model was exposed to a wide range of scenarios during training, which in turn facilitated in robust learning and improved performance on unseen data. Specifically class imbalance is addressed by ensuring sufficient representation of ship ports, ships, and containers of different sizes, orientations, and configurations. Data augmentation and resampling have increased the number of underrepresented classes and helped in balancing the class distribution throughout.

#### 3.1.4 Annotation

Annotation of the satellite images is performed using the ArcGIS tool to ensure precise interpretation of ship ports and ships within the satellite images. For this, the bounding boxes are drawn until the extent of ship ports and ships are detected accurately. Various types of ships and vessels present within the port area are annotated including cargo ships, container vessels, and oil tankers.

#### 3.1.5 Data pre-processing

This step includes cleaning the annotated dataset to remove artifacts, sensor noise, and irrelevant background elements. To ensure consistency across the dataset the resolution is standardized using MinMaxScaler.


(7) $$\begin{array}{l} {x^{\prime }}_{i}=\frac{x_{i}-x_{\min}}{x_{\max}-x_{\min}} \end{array}$$

Gabor (1946) Filter is employed to integrate texture analysis on the images to load more insights from them. This helps in extracting various hidden features from the ship and ship port scenes.

#### 3.1.6 Hyperparameter optimization

Enhanced YOLO is particularly designed to address various challenges the model may face during the detection process. During the model training process key hyperparameters were carefully optimized such as the learning rate was set to 0.001 and batch size was fixed to 16 inorder to balance the memory usage. Furthermore the IoU threshold was tuned to 0.5, to achieve an effective balance between precision and recall. These optimization techniques helped in improving the overall precision, recall and mAP metrics of the model at the end.

#### 3.1.7 Evaluation metrics

Various evaluation metrics were engaged in the process of assessing the performance of the ship and ship port model to test the effectiveness and accuracy of the algorithm.

• ***Precision***: The proportion of number of accurate detections to the number of detected ships and ports compared to total model detections is precision. This effectively identifies how well the model is identifying true positives while reducing false positives.


(8) $$\begin{array}{l} Precision\;=\;TP\;/\;\left(TP\;+\;FP\right) \end{array}$$

• ***Recall***: This proportion measures how well a model identifies ships and ship ports in relation to all ships and ship ports in the dataset.


(9) $$\begin{array}{l} Recall=TP/\;\left(TP+FN\right) \end{array}$$

• ***F1 Score***: The F1 score measures the harmonic average between precision and recall, thus giving an equal evaluation of performance on this model. It can be used to compare models with different tradeoffs between precision and recall because it combines both together into one metric.


(10) $$\begin{array}{l} F1\;score=\frac{\left(2\times Precision\times Recall\right)}{\left(Precision+Recall\right)} \end{array}$$

• ***Intersection over Union (IoU)***: IoU calculates the spatial overlapping of the predicted bounding boxes with the ground truth bounding boxes. This as a whole is evaluated as the ratio of intersection area to the total union area of the two bounding boxes, providing insights into the localization accuracy of the model.


(11) $$\begin{array}{l} IoU=\left(Area\;of\;Intersection\right)/\left(Area\;of\;Union\right) \end{array}$$

• ***Mean average precision (mAP)***: mAP measures the precision-recall evaluation curve for each category and generates the average precision, providing an overall assessment of the model's detection performance. It is calculated as the average precision spanning over different objects and their categories. This evaluates the precision and recall curve for each category of images and computes the average precision, providing an overall assessment of the model's detection performance.


(12) $$\begin{array}{l} mAP=\frac{1}{n}\sum_{i=1}^{n}AP_{i} \end{array}$$

## 4 Results and analysis

Various OpenCV libraries are employed to implement the proposed algorithms. The method for finding regions of interest (ROIs) was defined, with options for fast or quality detection. Selective Search is applied to the loaded dataset consisting of geospatial images to find the Region of Interests (ROIs). For ship detection, this method utilized a modified R-CNN model loaded from a saved file. This model is used to predict ROIs and a non-maximum variable suppression was applied to filter out overlapping predictions and employ their refinement. Bounding boxes were outlined around the detected ships, and the resulting images were displayed. Table 2 shows the model results for detection using R-CNN. Also, the post-augmentation techniques played a vital role in improving the model performance such as flipping, rotation, and scaling helped in generalizing the model across all scenarios, making it more comfortable even in unseen data.

**Table 2:** Model summary.

**Parameters**	**Value**
Number of classes	2
Learning rate	0.001
Epochs	50
Training samples	4,200
Validation samples	1,200
Testing samples	600
Optimizer	Adam
Accuracy (training)	0.9865
Accuracy (validation)	0.9917

The model is trained on Adam optimizer, with a batch size of 16 across 50 epochs. The training set achieved an accuracy of 0.9865 along with a validation set accuracy of 0.9917. The precision value for the “no-ship” class label was 0.99, and for that of the “ship” class label was 1.00. The recall for the “no-ship” class label was 1.00, and for the “ship” class label was 0.99.

The confusion matrix revealed that 1% of the “no-ship” samples were wrongly classified as “ship,” and 0.01% of the “ship” samples were misclassified as “no-ship.” When assessed in relation to the other models as in Table 3, the proposed method exhibits competitive performance across various metrics. For instance, Patel et al. (2022a) utilized YOLOv3, YOLOv4, and YOLOv5 models on the Airbus Ship Dataset, achieving a precision of 99%. Similarly Zhang et al. (2019) employed the Faster-R-CNN model on the GaoFen-2 dataset, achieving an accuracy of 96%. Still, there is no integration of port detection in any of the described approaches. The model performed well in detecting ships in satellite imagery. For the ship port detection, this method employed specifically an enhanced YOLO algorithm due to its highly accurate object detection. The same dataset is used for this and annotation, training, and gathering are performed to train the model effectively.

**Table 3:** Comparison with existing works.

**Method**	**Dataset**	**Methodology**	**Evaluation metrics**	**Accuracy**
Patel et al. (2022a)	Airbus ship dataset	YOLOv3, YOLOv4, YOLOv5	Precision	99%
Zhang et al. (2019)	GaoFen-2	Faster-R-CNN	Accuracy	96%
Patel et al. (2022b)	High-resolution satellite images dataset (HSRID)	YOLOv7	Adam optimizer	93%
Yu et al. (2016)	Google earth service	Hough forest	F1 score	97%
He et al. (2018)	TerraSAR-X SAR images	Adaptive component selection-based discriminative model (ACSDM)	Precision, recall	96.4%
Hwang et al. (2017)	X-band Kompsat-5 SAR	Artificial neural network (ANN)	Recall, precision	93.9%
Zhang and Zhang (2016)	High-resolution broad-area remote-sensing images	Two-layer saliency model with SVM	Precision	96%
Proposed model	Satellite images	Enhanced YOLO	Precision, recall, mAP50	84.5%

Table 4 provides an overview of evaluation metrics for port detection, focusing on precision, recall, mAP50 (mean Average Precision at 50%), and IOU (Intersection over Union). Specifically, in terms of precision, the proposed model achieved an impressive score of 0.832 for port detection, surpassing many existing approaches. This result showcased that the enhanced model gave an accurate prediction of port regions within the images with minimal false positive rate which is crucial in applications that require precise outlining of port boundaries. Over the gradual increase of epochs, the model is able to capture significant portions of port instances with a commendable score of 0.633. This indicated the model's effectiveness in the accurate detection of ports across various scales of regions and contexts. Furthermore, the mAP50 score of 0.702 highlights the model's proficiency in accurately varying spatial overlap degrees between ground truth and bounding predicted boxes. In contrast to existing models, the proposed approach stands out for its unique and accurate identification of port areas amidst various maritime environments.

**Table 4:** Results of the proposed system for port detection.

**Class**	**Images**	**Instances**	**Precision**	**Recall**	**mAP50**	**IOU**
All	26	134	0.67	0.514	0.572	0.456
Port	26	30	0.832	0.633	0.702	0.609
Ship	26	104	0.509	0.394	0.443	0.302

**Figure 5:**
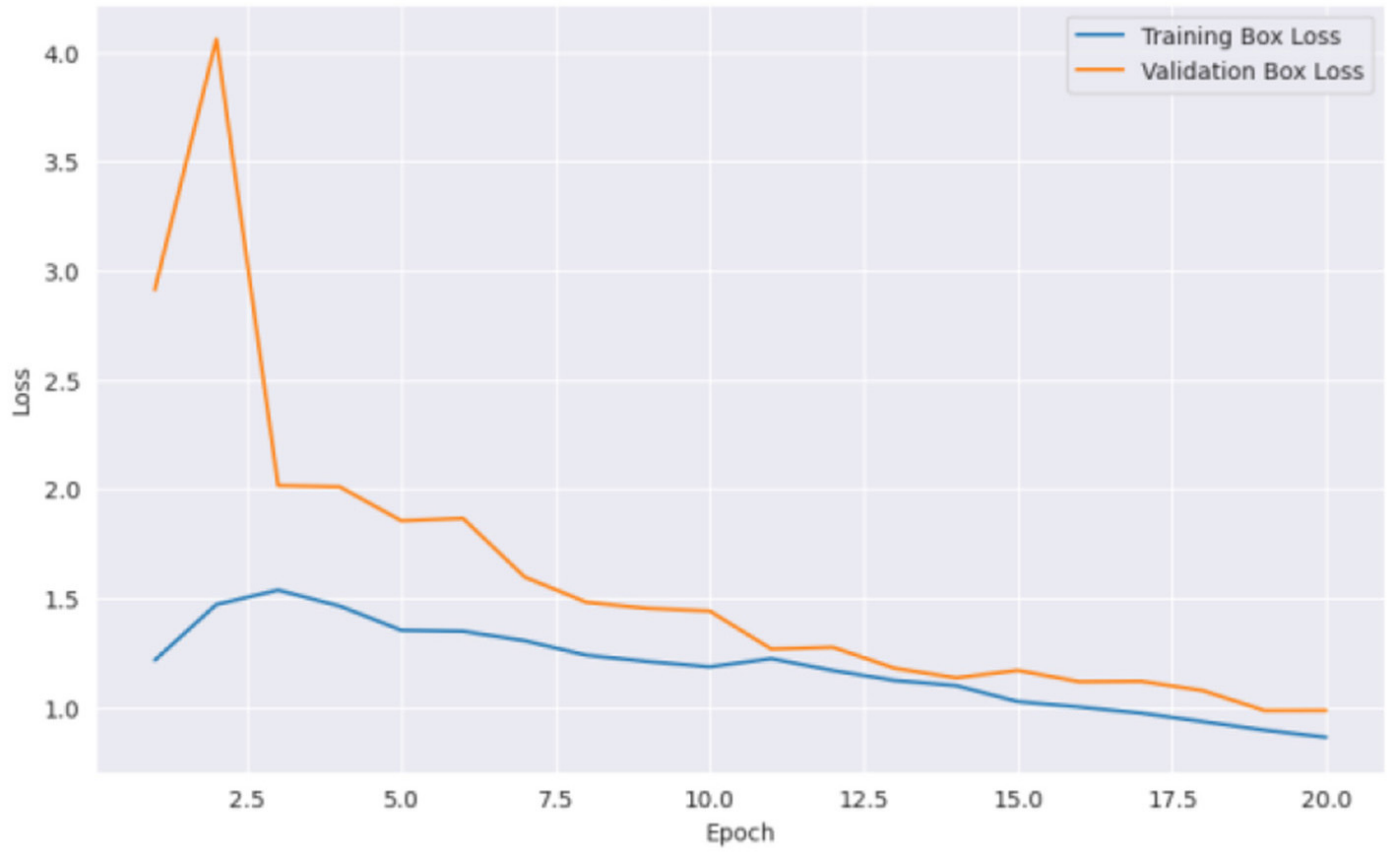
Epochs vs. loss.

**Figure 6:**
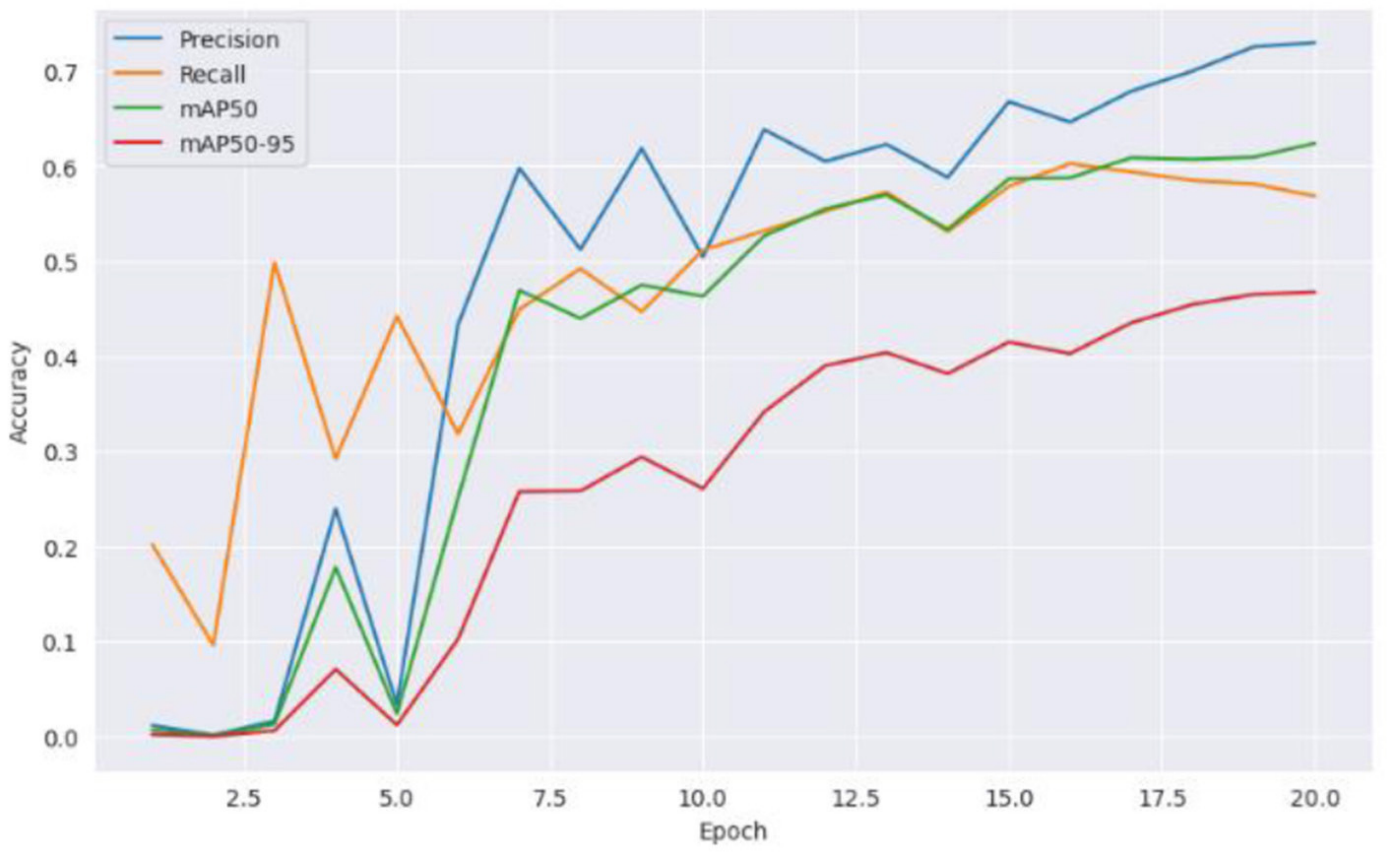
Accuracy vs. epochs.

**Figure 7:**
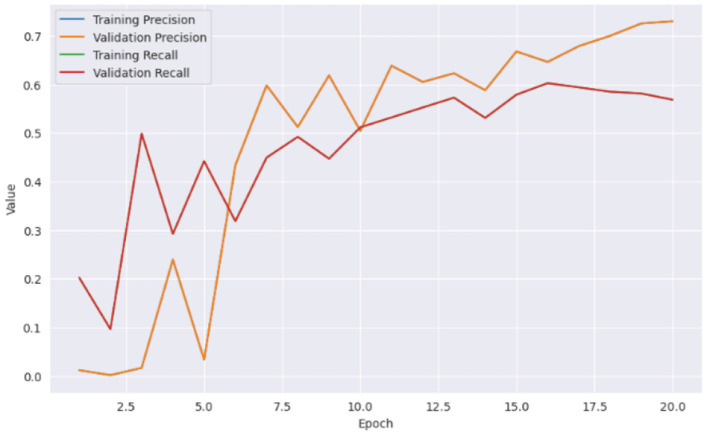
Training and validation metrics.

From Figure 5 it can be inferred that the proposed model performed well on the training data and generalizing over unseen data. This observed loss curve behavior depicted that the model continued to improve over the increase of epochs. In Figure 6, both training and validation metrics showed positive trends over epochs. Although there is a minor decrease in training recall, the model's overall performance is encouraging, as indicated by the increasing validation recall. Figure 7 further supported that the positive trend showed a steady increase in accuracy as the number of epochs increased.

Overall, the proposed approach offers a unique task in the detection of ports providing a reliable and efficient solution offering high precision, recall, mAP50, and IOU scores. All these results These capabilities position our model as a robust tool for maritime surveillance, port management, and related applications, contributing to enhanced efficiency and effectiveness in monitoring and safeguarding maritime infrastructure.

In evaluating the ship detection models, R-CNN and enhanced YOLO showcased commendable performance not only in ship detection but also in ship port detection which is the unique identity of the proposed method. For R-CNN, precision and recall values of 0.94 and 0.89 were achieved, respectively, along with an accuracy of 92% on the validation set, affirming its proficiency in ship classification including ship port areas. On the other hand, YOLO exhibited robustness with a precision of 0.908, recall of 0.888, mAP50 of 0.952, and mAP50–95 of 0.692, demonstrating its efficacy in accurately detecting ships and their port areas. These models ensure a balance between precise classification and comprehensive coverage, encompassing both ships and their surrounding port environments.

In the process of implementation, various box loss values were calculated to assess the models performance such as training box loss, class loss, etc. Additionally, various evaluation metrics such as precision, recall, and mAP50 gave variable insights into the models effectiveness not only in ship detection but also in ship port detection.

**Figure 8:**
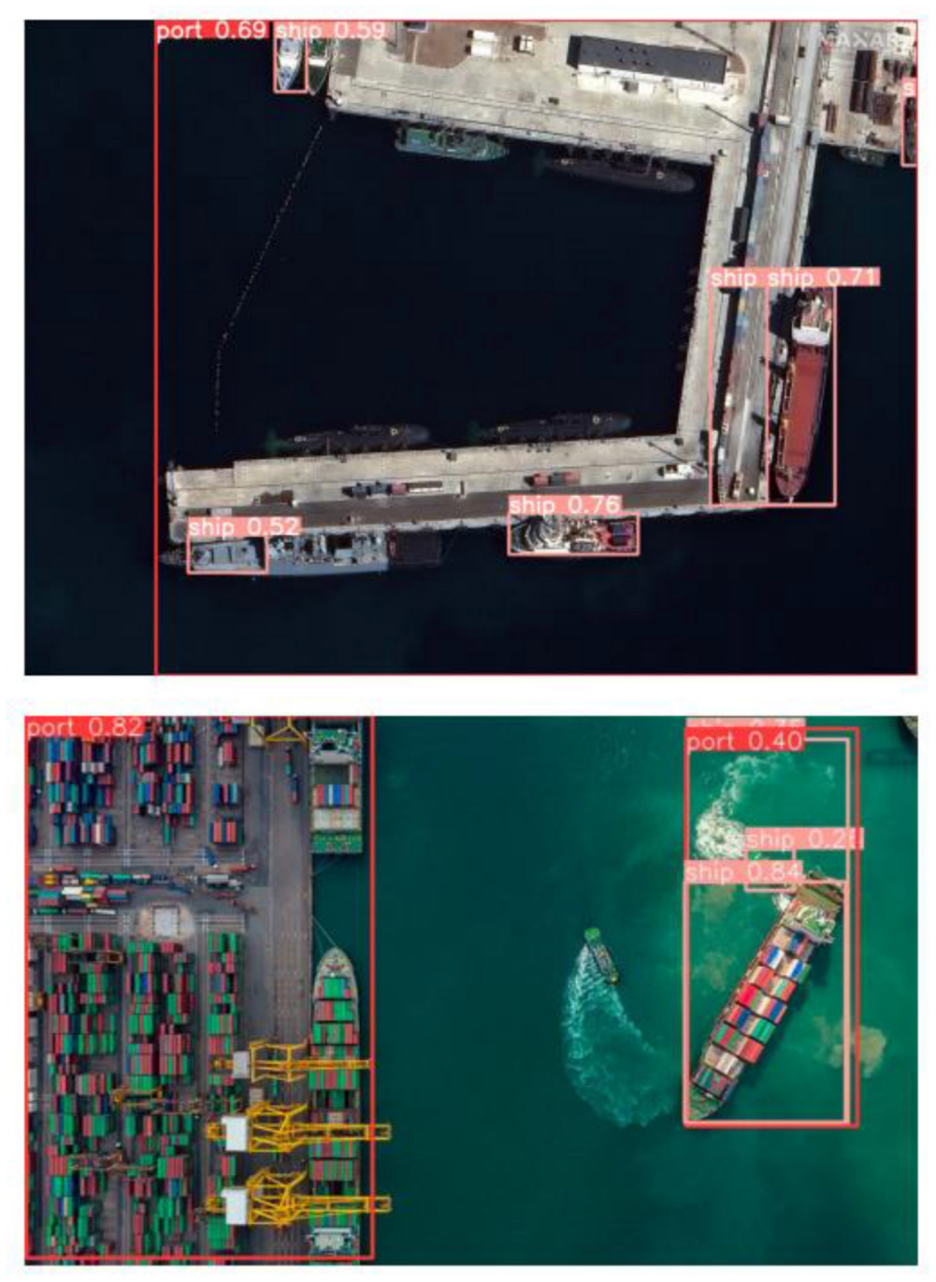
Result of ship port detection.

Moreover, the models' generalization capability and its effective performance on the real-world data are evaluated on the unseen data as in Figure 8. This included various post-processing techniques such as thresholding for refined predictions and non-maximum suppression on both ship and port areas. This effective approach enabled a thorough analysis of the models effectiveness in various marine environments.

### 4.1 Implementation of the proposed approach on Jetson Nano

NVIDIA developed the Jetson Nano computer module, for edge AI applications offering a cost-compact solution. With its GPU architecture to that of other devices, it excels in executing deep learning algorithms. Industries like robotics, drones, IoT devices, and embedded systems commonly utilize it for AI processing without relying on cloud services. It serves as a platform for developers and enthusiasts to experiment develop and deploy AI applications in a compact form factor.

Getting YOLO set up for object detection on the Jetson Nano involves some steps. First, install required libraries like PyTorch and download trained YOLO weights. Create an environment to avoid any conflicts before running Python to load the model and prepare the input image for analysis. The real magic happens during inference when YOLO identifies objects. Finally convert the model output into a format such as bounding boxes overlaid on an image. For optimized performance, on the Jetson Nanos resources consider using a version of the advanced YOLO model or implementing quantization techniques for quicker processing speed.

To create a more straightforward model that could be installed on the Jetson Nano and tuned for object detection, the proposed model used optimization techniques with TensorRT. The YOLO model is first changed to a lighter version that is more suited for the Jetson Nano's limited resources using PyTorch's torch.onnx.export() function. As a result, TensorRT is compatible with the model. After TensorRT is installed on the development system, it optimizes the ONNX model for Jetson Nano GPU inference. TensorRT performs improvements such as layer fusion, accurate calibration, and kernel auto-tuning to improve efficiency. Additionally, several quantization techniques are applied to reduce the model's size and expedite inference. Both dynamic range and integer quantization are supported by TensorRT, which lowers the model's weights while guaranteeing faster processing with little degradation of accuracy. Lastly, the Jetson Nano is used to assess the performance of the optimized model.

After analyzing the data from Table 5, it concluded that the system performs 2.2 times faster when operating in “MaxN” mode compared to the 5 W mode. This difference is likely due to the system being able to draw power in “MaxN” mode leading to performance. On the hand carrying out inference tasks takes longer in low power settings, like the 5W mode.

**Table 5:** Inference in different scenarios.

**Power consumption**	**Inference time**
5 W	36,298.4 ms
MaxN	16,434.2 ms

## 5 Conclusions and future work

The proposed approach demonstrates the effectiveness of the enhanced YOLO in ship and port detection. It emphasized its potential uses for real-world maritime security and surveillance. The current approach uses the Jetson Nano's CPU for object detection with YOLO and achieves a reasonable inference speed, there is still potential for improvement. Additional monitoring is required for assessing the GPU utilization. Moving forward, various techniques can be employed to further optimize the proposed YOLO for faster inference speed on Jetson Nano. Further improvements could explore smaller YOLO model variants to optimize accuracy. Quantization approaches can minimize model size while increasing processing speed on the Jetson Nano hardware.

Fine-tuning the model can eliminate extraneous components and use code profiling to identify the bottlenecks to further improve the performance. Various libraries like TensorRT can be used for inference optimization. Carrying out these methods and resource monitoring can greatly reduce the inference time while ensuring that the proposed model runs effectively on the Jetson Nano.

## Data Availability

The original contributions presented in the study are included in the article/supplementary material, further inquiries can be directed to the corresponding author.
